# A nomogram for individualized prediction of overall survival in patients with newly diagnosed glioblastoma: a real-world retrospective cohort study

**DOI:** 10.1186/s12893-021-01233-z

**Published:** 2021-05-06

**Authors:** Nijiati Kudulaiti, Zhirui Zhou, Chen Luo, Jie Zhang, Fengping Zhu, Jinsong Wu

**Affiliations:** 1 Department of Neurosurgery, Huashan Hospital, Shanghai Medical College, Fudan University, Shanghai, China; 2 Neurosurgical Institute of Fudan University, Shanghai, China; 3 Shanghai Clinical Medical Center of Neurosurgery, Shanghai, China; 4 Shanghai Key Laboratory of Brain Function and Restoration and Neural Regeneration, Shanghai, China; 5 Radiation Oncology Center, Huashan Hospital, Shanghai Medical College, Fudan University, Shanghai, China

**Keywords:** Glioblastoma, Nomogram, Lasso-Cox regression, Prognosis

## Abstract

**Background:**

This study aimed to identify the most valuable predictors of prognosis in glioblastoma (GBM) patients and develop and validate a nomogram to estimate individualized survival probability.

**Methods:**

We conducted a real-world retrospective cohort study of 987 GBM patients diagnosed between September 2010 and December 2018. Computer generated random numbers were used to assign patients into a training cohort (694 patients) and internal validation cohort (293 patients). A least absolute shrinkage and selection operator (LASSO)-Cox model was used to select candidate variables for the prediction model. Cox proportional hazards regression was used to estimate overall survival. Models were internally validated using the bootstrap method and generated individualized predicted survival probabilities at 6, 12, and 24 months, which were compared with actual survival.

**Results:**

The final nomogram was developed using the Cox proportional hazards model, which was the model with best fit and calibration. Gender, age at surgery, extent of tumor resection, radiotherapy, chemotherapy, and IDH1 mutation status were used as variables. The concordance indices for 6-, 12-, 18-, and 24-month survival probabilities were 0.776, 0.677, 0.643, and 0.629 in the training set, and 0.725, 0.695, 0.652, and 0.634 in the validation set, respectively.

**Conclusions:**

Our nomogram that assesses individualized survival probabilities (6-, 12-, and 24-month) in newly diagnosed GBM patients can assist healthcare providers in optimizing treatment and counseling patients.

*Trial registration*: retrospectively registered.

## Background

Gliomas are among the most common primary brain tumors in adults and account for over 81% of malignant brain tumors. Glioblastoma (GBM), the most malignant type, accounts for the majority of gliomas (56.6%) and has an incidence of 3.21 per 100,000 [1]. GBM patients have a poor prognosis. Five-year relative survival is 5.6% and median overall survival (OS) is 12 to 15 months [1]. Factors affecting prognosis include age, sex, Karnofsky performance status (KPS), extent of resection (EOR), treatment plan, and several biomarkers [2–8]. These biomarkers include isocitrate dehydrogenase enzyme (IDH) mutation, telomerase reverse transcriptase (TERT), O6-methylguanine-DNA methyltransferase (MGMT) gene promoter methylation status and epidermal growth factor receptor (EGFR) [5–9]. After maximal safe resection and subsequent concurrent chemoradiation and adjuvant chemotherapy with the alkylating agent temozolomide, median survival is still < 2 years [3].

Nomograms are accessible tools that physicians can use to predict survival, make treatment decisions based on individualized cancer prognosis and create follow-up plans. Several nomograms have been previously developed for GBM patients [10–12]. One was developed using data from the European Organization for Research and Treatment of Cancer-National Cancer Institute of Canada clinical trial; however, this nomogram was only internally validated [10]. Another nomogram, which was both internally and externally validated, was developed from data from two independent, nonoverlapping NRG Oncology Radiation Therapy Oncology Group (RTOG) clinical trials (0525 and 0825) [11]. The analysis for this nomogram included only patients who completed concurrent chemoradiation from both trials and therefore several important treatment-related prognostic factors, such as IDH mutation status and use of concurrent chemoradiation therapy were not considered. In addition, another recent study developed a nomogram to estimate individualized survival probabilities for newly diagnosed IDH-wild-type GBM patients using data from the Ohio Brain Tumor Study (OBTS) that was externally validated using data from the University of California San Francisco [12].

The purpose of this study was to identify the most valuable prognostic indicators from real-world clinical data, then develop and validate a readily accessible and practical nomogram that estimates individualized survival probability for GBM patients.

## Methods

### Study population and design

A total of 987 GBM patients diagnosed between September 2010 and December 2018 at Huashan Hospital (an affiliate of Fudan University) were retrospectively enrolled in this cohort study. Computer generated random numbers were used to assign patients to a training cohort (n = 694) and an internal validation cohort (n = 293). This study was approved by the ethics review committee of Huashan Hospital, and written informed consent was obtained from all individual participants included in the study.

### Data collection

Histological diagnosis of GBM was based on specimens obtained during surgical resection. Haematoxylin–eosin stained sections of all specimens were reviewed by two blinded neuropathologists and classified according to the 2016 World Health Organization Classification of Tumors of the Central Nervous System. The following variables were obtained for each patient: gender, age at surgery, KPS score before surgery, number of days in hospital, tumor location, EOR, number of operations, tumor laterality, IDH1 status, MGMT status, TERT status, Ki67 index, radiotherapy, chemotherapy, adjuvant therapy, and recurrence and survival status. IDH1 testing was performed by immunohistochemistry. Standard pyrosequencing was performed to test MGMT methylation. All testing results were reviewed by an expert neuropathologist. Tumor recurrence was determined using the Response Assessment in Neuro-Oncology criteria.

### Statistical analyses

The t-test and Chi-square test were used to compare continuous variables and categorical variables, respectively, between the two datasets. A penalized Cox model was applied to select variables for constructing a predictive model. The R package glmnet was used to apply the least absolute shrinkage and selection operator (LASSO) to the model. After cross-validation methods were used to test the robustness of the selected significant candidate characteristics, the model was used to weight the coefficients from the training cohort to build the prediction model [13].

Based on the results from these variables, patients in the training and validation datasets were classified into high-risk score and low-risk score groups. The Kaplan–Meier method was used to calculate OS in each dataset and the log-rank test was used to compare the difference. Cox proportional hazards (CPH) regression was used to assess OS. The models were trained using the training set and internally validated using the test set. The bootstrap method was used to internally validate the models to generate individual predicted survival probabilities at 6, 12, 18, and 24 months, which were compared with observed actual survival to measure prediction accuracy. The CPH OS prediction model was evaluated by the concordance index, which ranges from 0.5 (completely random prediction) to 1 (perfect prediction). The final nomogram was developed using the Cox method with the greatest prediction accuracy to individualize estimated survival probability. Calibration curves were also drawn for each dataset. All analyses were performed using R version 3.6.3. P < 0.05 was considered significant.

## Results

### Patient characteristics

Patient demographics for all patients (N = 987) and the training (N = 694) and validation datasets (N = 293) are presented in Table 1. There were no significant differences in any of the measured variables between the training and validation datasets.

**Table 1:** Baseline characteristics of the glioblastoma patients overall and in the training and validation datasets

Variable	Overall (N = 987)	Training set (N = 694)	Validation set (N = 293)	P-value
Gender [N (%)]				
Female	365 (37.0)	265 (38.2)	100 (34.1)	0.257
Male	622 (63.0)	429 (61.8)	193 (65.9)	
Age_at_surgery [mean (SD)]	52.60 (14.12)	52.89 (13.62)	51.91 (15.23)	0.322
KPS score before surgery [mean (SD)]	85.53 (8.73)	85.50 (8.80)	85.62 (8.57)	0.846
Days_in_hospital [mean (SD)]	18.99 (9.35)	18.79 (9.75)	19.46 (8.36)	0.338
Surgical_resection [N (%)]				
Total resection	620 (80.7)	438 (81.1)	182 (79.8)	0.67
Subtotal resection	138 (18.0)	94 (17.4)	44 (19.3)	
Partial resection	10 (1.3)	8 (1.5)	2 (0.9)	
Number_of_operations [N (%)]				
1	810 (94.0)	571 (94.2)	239 (93.4)	0.093
2	50 (5.8)	35 (5.8)	15 (5.9)	
3	2 (0.2)	0 (0.0)	2 (0.8)	
Laterality [N (%)]				
Left	383 (48.7)	261 (47.1)	122 (52.6)	0.186
Right	403 (51.3)	293 (52.9)	110 (47.4)	
Location [N (%)]				
Callosum	43 (5.2)	26 (4.5)	17 (6.9)	0.419
Frontal lobe	368 (44.3)	267 (45.8)	101 (40.7)	
Parietal lobe	87 (10.5)	65 (11.1)	22 (8.9)	
Temporal lobe	267 (32.1)	178 (30.5)	89 (35.9)	
Occipital lobe	33 (4.0)	25 (4.3)	8 (3.2)	
Insular lobe	27 (3.2)	18 (3.1)	9 (3.6)	
Cerebellum	6 (0.7)	4 (0.7)	2 (0.8)	
IDH1 status [N (%)]				
Wild-type	680 (91.3)	471 (91.3)	209 (91.3)	1
Mutant-type	65 (8.7)	45 (8.7)	20 (8.7)	
Ki-67 index [N (%)]				
Less than 5%	38 (4.0)	27 (4.0)	11 (3.9)	0.568
5–20%	559 (58.7)	401 (59.8)	158 (56.2)	
More than 20%	355 (37.3)	243 (36.2)	112 (39.9)	
MGMT status [N (%)]				
Unmethylated	457 (61.0)	323 (61.8)	134 (59.3)	0.58
Methylated	292 (39.0)	200 (38.2)	92 (40.7)	
TERT status [N (%)]				
Wild-type	85 (43.6)	57 (41.9)	28 (47.5)	0.575
Mutant-type	110 (56.4)	79 (58.1)	31 (52.5)	
Radiotherapy [N (%)]				
No	192 (19.5)	136 (19.6)	56 (19.1)	0.93
Yes	795 (80.5)	558 (80.4)	237 (80.9)	
Chemotherapy [N (%)]				
No	219 (22.2)	160 (23.1)	59 (20.1)	0.355
Yes	768 (77.8)	534 (76.9)	234 (79.9)	
Adjuvant therapy [N (%)]				
Radiotherapy and chemotherapy	728 (73.8)	508 (73.2)	220 (75.1)	0.619
Radiotherapy only	67 (6.8)	50 (7.2)	17 (5.8)	
Chemotherapy only	39 (4.0)	25 (3.6)	14 (4.8)	
No adjuvant therapy	153 (15.5)	111 (16.0)	42 (14.3)	
Recurrence status [N (%)]				
No recurrence	174 (17.6)	129 (18.6)	45 (15.4)	0.261
Recurrence	813 (82.4)	565 (81.4)	248 (84.6)	
Survival status [N (%)]				
Alive	221 (22.4)	161 (23.2)	60 (20.5)	0.393
Dead	766 (77.6)	533 (76.8)	233 (79.5)	

*KPS* Karnofsky performance status, *IDH1* isocitrate dehydrogenase 1, *MGMT* O6-methylguanine-DNA methyltransferase, *TERT* telomerase reverse transcriptase

### Feature selection and risk score building

**Fig. 1:**
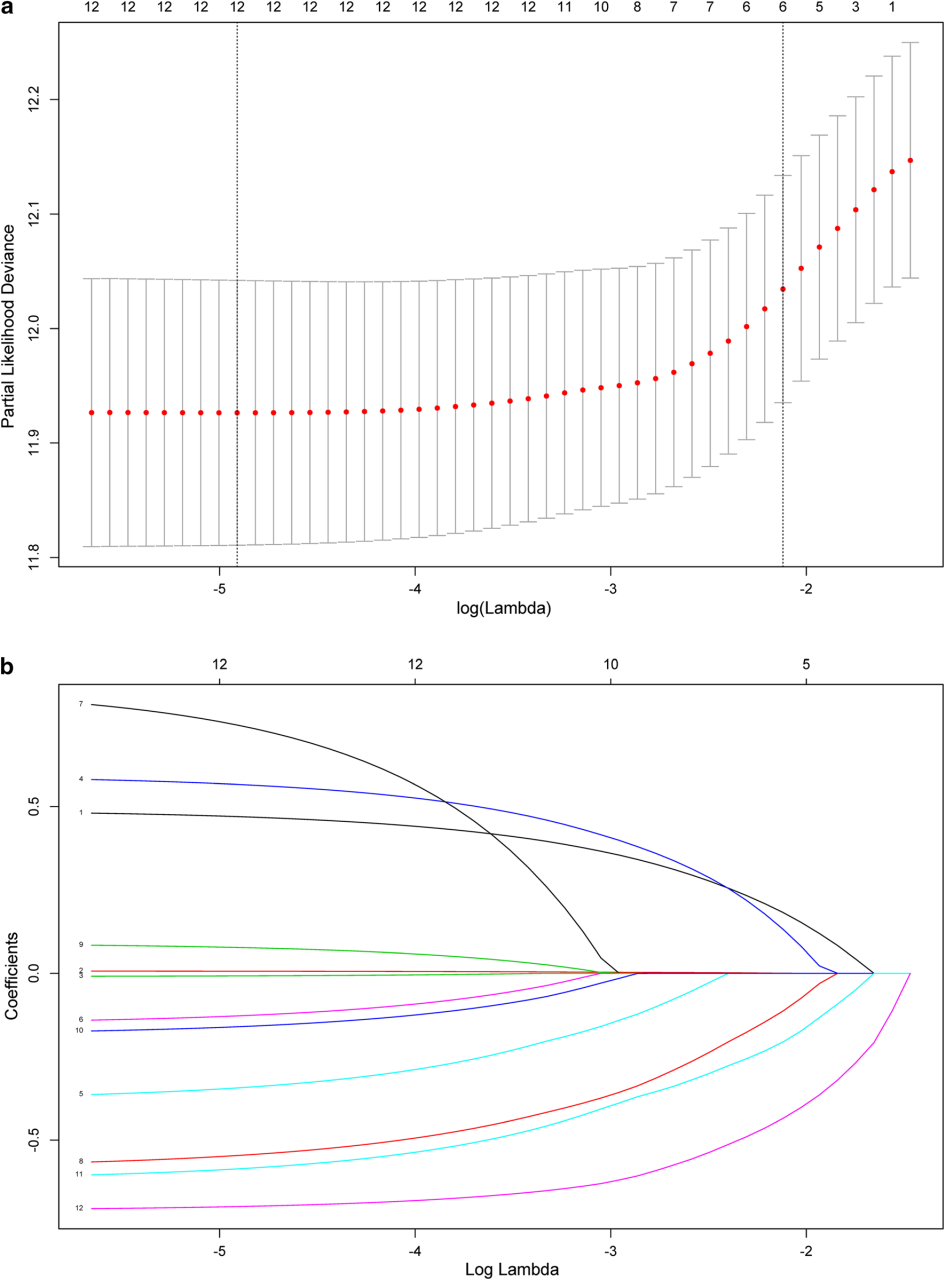
Prognostic factor selection using the least absolute shrinkage and selection operator (LASSO) Cox regression model. **a** Tuning parameter (λ) selection in the LASSO model used tenfold cross-validation via minimum criteria. The partial likelihood deviance curve was plotted versus log(λ). Dotted vertical lines were drawn at the optimal values by using the minimum criteria and the one standard error of the minimum criteria (the 1-SE criteria). A λ value of 0.1201, with log (λ), − 2.1193 was chosen (1-SE criteria) according to tenfold cross-validation. **b** LASSO coefficient profiles of the 12 prognostic factors. A coefficient profile plot was produced against the log (λ) sequence. A vertical line was drawn at the value selected using tenfold cross-validation, where optimal λ resulted in six nonzero coefficients

In terms of prognostic factors, 12 features were reduced to 6 potential predictors based on 374 patients in the training set (Fig. 1a, b), which were features with nonzero coefficients in the LASSO-Cox model. These features are presented in the risk score calculation formula:

$${\text{Risk score }} = \, 0.1822 \times {\text{gender}} + 0.0002 \times {\text{age at surgery}} + 0.1320 \times {\text{surgical resection}} - 0.1133 \times {\text{IDH}}1{\text{ status}} - 0.2062 \times {\text{radiotherapy}} - 0.4353 \times {\text{chemotherapy}}$$

### Development of an individualized prediction model

Cox regression analysis identified gender, age at surgery, EOR, IDH1 status, radiotherapy, and chemotherapy as independent predictors (Table 2). The final concordance index of the CPH model was 0.635. In the multivariable CPH analyses of the two datasets, female gender, radiotherapy, chemotherapy, IDH1 mutant and total resection were significantly associated with better OS (all P < 0.05). Younger age at time of surgery trended toward better OS but was not statistically significant (P = 0.088). There was a significant OS difference between total resection and partial resection (P = 0.027), but not between total resection and subtotal resection (P = 0.785).

**Table 2:** Cox proportional hazards model results from the training set

Variable	HR	95% CI	Wald Z	P-value
Gender (Male vs. female)	1.309	(1.033–1.659)	2.229	0.026
Age at surgery (< 55 years vs. > 55 years)	1.008	(0.999–1.018)	1.705	0.088
Surgical resection (Partial vs. Total)	2.242	(1.098–4.579)	2.215	0.027
Surgical resection (Subtotal vs. Total)	1.066	(0.674–1.686)	0.272	0.785
IDH1 status (Mutant vs. wild-type)	0.489	(0.3116–0.768)	− 3.108	0.002
Radiotherapy (Yes vs. No)	0.675	(0.454–1.002)	− 1.949	0.051
Chemotherapy (Yes vs. No)	0.505	(0.347–0.734)	− 3.576	0.000

*IDH1* isocitrate dehydrogenase 1

**Fig. 2:**
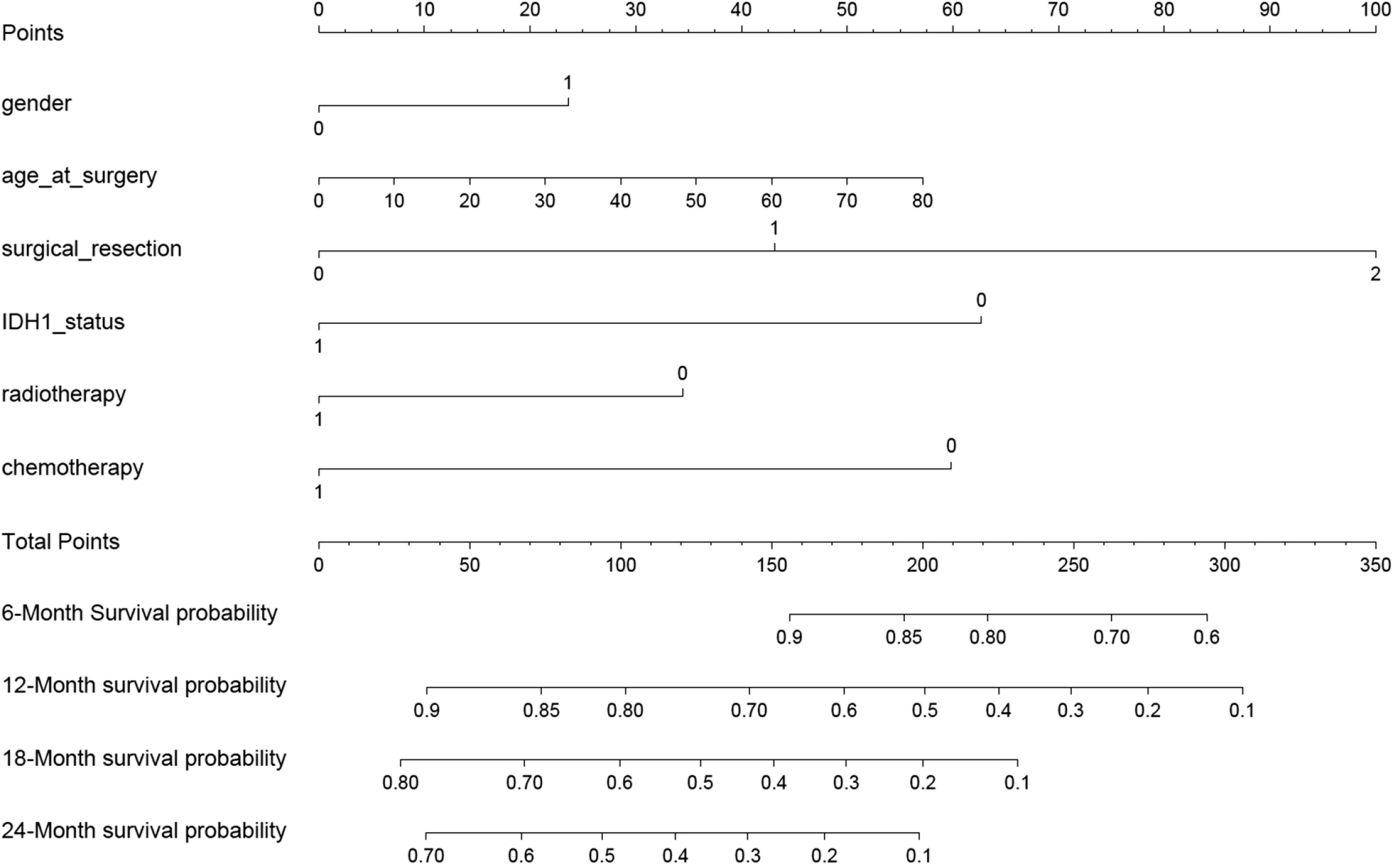
Nomogram for predicted 6-, 12-, and 24-month survival probabilities in glioblastoma patients. Gender (1 = male, 0 = female); age_at_surgery: age at the time of surgery; surgical_resection: status of surgical excision (0 = gross total resection, 1 = subtotal resection, 2 = partial resection); IDH1_status: IDH1 gene mutation status (0 = wild-type, 1 = mutant); radiotherapy: receipt of radiation therapy (1 = yes, 0 = no); chemotherapy: receipt of chemotherapy (1 = yes, 0 = no)

The model that incorporated the above independent predictors was developed and is presented as the nomogram (Fig. 2). The nomogram to estimate 6-, 12-, and 24-month survival probabilities was established using the training and validated dataset using the CPH model.

### Survival

**Fig. 3:**
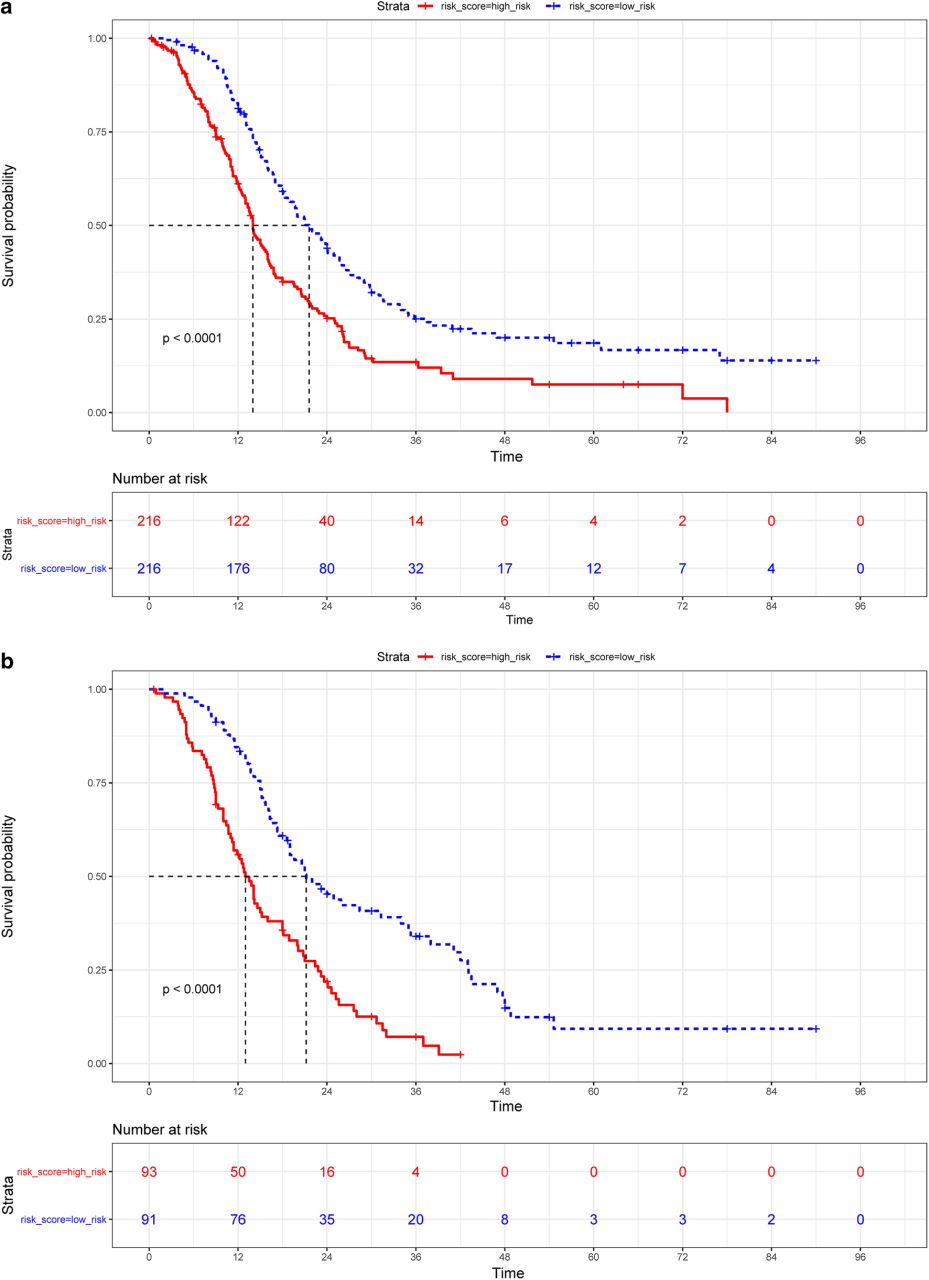
Kaplan–Meier survival curves for glioblastoma patients. **a** Training dataset and **b** validation dataset

Patients in the training dataset were divided into high-risk score and low-risk score groups. Kaplan–Meier curves were generated for each group and median survival rates with 95% confidence intervals (CIs) were calculated (Fig. 3). In the high-risk score group, median survival was 14.0 months (95% CI 13.0–15.9). In the low-risk score group, median survival was 21.6 months (95% CI 19.0–24.9). The difference was significant (P < 0.0001, Fig. 3a). In the validation dataset, median survival also significantly differed between the two groups (13.0 months (95% CI 11.2–16.0) vs. 21.2 months (95% CI 19.0–35.0); P < 0.0001; Fig. 3b).

### Model discrimination validation

**Fig. 4:**
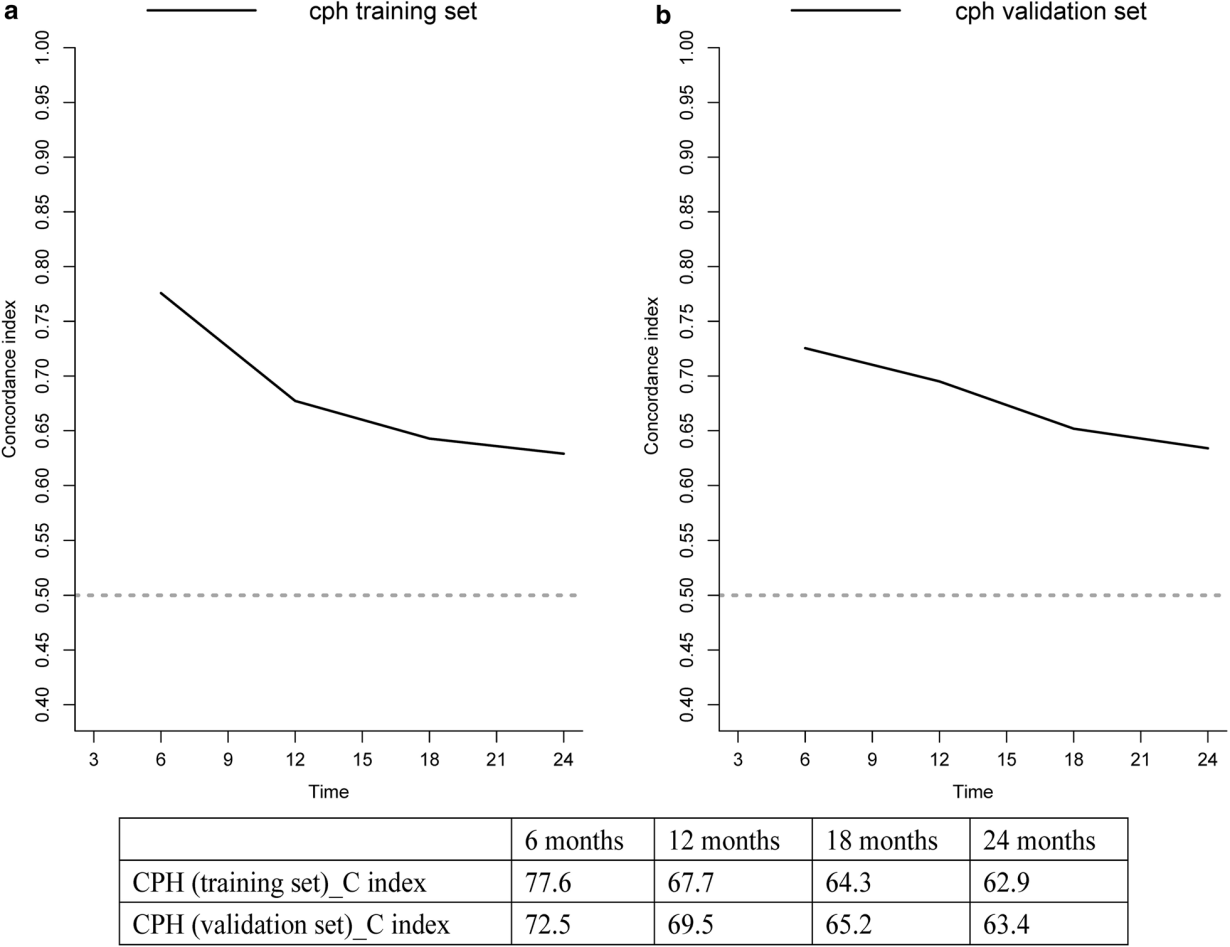
Concordance indices of the Cox proportional hazard model. Concordance indices of the Cox proportional hazard model at 6, 12, 18, and 24 months in the training dataset (**a**) and validation dataset (**b**)

After tenfold cross-validation of the training and validation datasets, the concordance index of each dataset was computed to predict survival at 6, 12, 18, and 24 months. For the respective 4 time points, the CPH analysis results of the training dataset were 0.776, 0.677, 0.643, and 0.629; those for the validation dataset were 0.725, 0.695, 0.652, and 0.634. Concordance index curves for the predicted 6-, 12-, 18-, and 24-month OS rates of the training and validation datasets are illustrated in Fig. 4 for a visual comparison.

### Model calibration validation

**Fig. 5:**
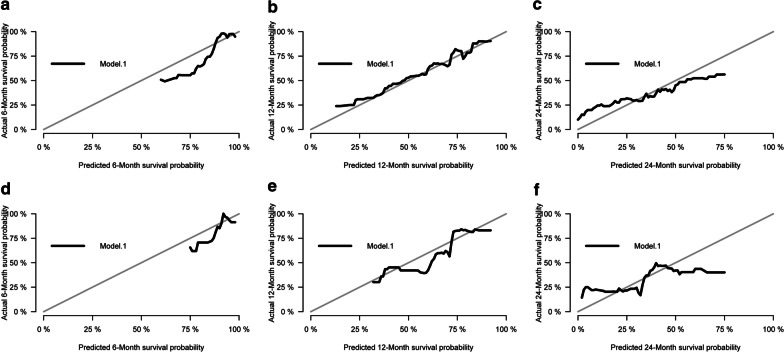
Calibration curves for survival probability. Calibration curves for survival probability at 6, 12, and 24 months in the training (**a**–**c**) and validation (**d**–**f**) datasets. The black line shows the observed survival probabilities versus the predicted probabilities and the grey line shows the ideal prediction

We also constructed calibration curves of the training and validation datasets to visually compare the predicted survival at 6, 12, and 24 months (Fig. 5a–f). For the predicted survival curves, the observed, and ideal survival rates lines are essentially identical, which suggests that the model’s predictions are in line with expectations. For the predicted 12-month survival curves (Fig. 5b, e), the observed and ideal lines essentially overlap.

## Discussion

In this real-world cohort study, we identified the most valuable prognostic indicators for patients with GBM, and then developed and validated an individual survival nomogram. According to bootstrap validation, the CPH survival model was the model with best fit and calibration. This model was then internally validated.

The most decisive factors in GBM prognosis are age at diagnosis, EOR and KPS [2, 14]. In our study, we considered up to 15 prioritized clinical features for each patient as possible prognostic factors. Using LASSO-Cox analysis, we found the six most valuable variables, namely gender, age at surgery, EOR, radiotherapy, chemotherapy, and IDH1 mutation status. These variables are easily acquired from patients, allowing easy application of the model in real-world practice. The incidence of GBM is 1.6 times higher in males compared to females [1] and 5-year cancer-specific survival rates in males and females are 6.8% and 8.3%, respectively [15]. Multivariable Cox proportional hazards models for patients with newly diagnosed IDH wild-type GBM from the OBTS showed a hazard ratio of 1.596 when comparing males with females (P = 0.011); however no significant difference was found when using data from University of California San Francisco (hazard ratio 1.206, P = 0.402) [12]. In another study that used data from the NRG Oncology RTOG clinical trial 0525, the hazard ratio was 1.596 (P = 0.0014) [11]. Our results are in agreement: females have a significantly better survival outcome.

Increased age is related to shorter survival [1] and GBM patients older than 75 years have a significantly higher risk of death than those aged 65–69 years [16]. Poorer survival in elderly GBM patients is due to coexisting disease as well as decreased ability to withstand neurological damage caused by the tumor, surgery and/or adjuvant therapy [15, 17, 18]. In addition, primary GBM and genes associated with poorer prognosis are more common in older patients [1, 6]. As in previous nomogram studies, [11, 12] we also found that age was an important predictor of prognosis.

Many studies have confirmed the importance of aggressive surgical resection when feasible. The prognosis of GBM patients with a greater EOR tends to be better, as maximum resection volume is associated with longer progression-free survival (PFS) and OS [19–22]. One previous study retrospectively analyzed 416 newly diagnosed and recurrent GBM patients and concluded that > 98% resection is necessary to significantly improve survival [2]. Multiple other studies have confirmed this, proving that OS in GBM patients is associated with greater EOR, [23] even in elderly patients, who are considered to have poorer outcomes regardless of intervention [24, 25]. A large study of 500 newly diagnosed GBM patients demonstrated that even EOR as low as 78% is related to improved OS, and when EOR exceeds 78%, OS continues to increase with the increase in EOR [26]. In a retrospective systematic meta-analysis of more than 41,000 newly diagnosed GBM patients, gross total resection was superior to subtotal resection, showing increases of 61% and 51% in 1-year OS and PFS, respectively [27]. Another study found that even 70% resection resulted in significant improvement in OS and seizure control [7]. However, although we found that EOR was an important predictor of prognosis, our analysis showed a significant difference only between total and partial resection, not between total and subtotal resection. The same results have been found in studies that used other databases [11, 12].

IDH1 mutations in GBM were first reported by Parsons et al. in 2008, [28] who pointed out that “mutations in IDH1 occurred in a large fraction of young patients and in most patients with secondary glioblastomas and were associated with an increase in OS.” Although IDH mutations can be found in up to 80% of grade II–III gliomas and secondary GBMs, they are rare in primary GBMs [6, 29–31]. GBMs are divided into three subgroups according to IDH mutation status: mutant, wild-type and not otherwise specified (NOS) [6, 9, 32, 33]. One analysis of GBM patients who underwent surgery and radiotherapy showed that mean OS in IDH1 mutant patients was 27.1 months, while mean OS in IDH1 wild-type patients was only 11.3 months [31]. Another study of GBM patients treated with radio/chemotherapy found that mean OS was 31 months in IDH1 mutant patients, which was twice that of IDH1 wild-type [30]. The role of IDH1 mutation as a predictor of prognosis was not considered in studies prior to these. In our study, IDH1 was found to be a more important predictor than MGMT and TERT. In our model, MGMT does not become one of the predictive model variables, however, in previous studies, MGMT became a predictive variable, which may be caused by the data in previous studies [11, 12]. In the study based on the NRG Oncology RTOG Clinical Trial 0525 database, there was no IDH mutation status in the model because no information related to IDH mutation status was provided in the database [11]. In another study that used data from Ohio Brain Tumor Study and University of California San Francisco, only the data of IDH wild-type patients were used, so the weight of IDH mutation status on prognosis was not reflected. [12] When our model was established, we did not artificially select the variables needed for prediction. Instead, we used LASSO to reduce the dimension of clinical variables. In this process, MGMT in real world data did not show the same results as other studies.

Because of the highly malignant nature of GBM, postoperative radiotherapy and chemotherapy are usually required. Our study found that postoperative radiotherapy and chemotherapy are also important predictors of prognosis. Radiotherapy can be used as either primary treatment or post-operatively and both can improve PFS and OS [34, 35]. Temozolomide administration with radiotherapy significantly increases OS in patients with newly diagnosed GBM from 12.1 months with radiotherapy alone to 14.6 months with radiotherapy and temozolomide [36]. Although radiation in combination with temozolomide is recommended over single-modality therapy for newly diagnosed GBM patients who are older than 70 years of age and have good performance status, the results of two phase III studies support the recommendation that temozolomide alone as initial therapy may be a reasonable option for elderly patients who have MGMT promoter-methylated tumors and would be initially preferred to delayed radiation treatment [37, 38].

Since our study is a real-world retrospective cohort study, it reflects problems encountered in actual clinical practice better than previous studies based on data from specific clinical databases. The nomogram study used to estimate individualized survival probability of GBM patients based on the RTOG database may not be suitable for GBM patients who do not meet their study criteria [12]. In addition, although radiotherapy and chemotherapy after maximal safe tumor resection is optimal, this treatment approach may not apply to all patients in the real world for various reasons. Using EOR, radiotherapy and chemotherapy as separate predictive model variables is more applicable in actual practice. Furthermore, the variables used in our prediction model were screened by LASSO regression, not set in advance, which is more objective.

There are several limitations to this study. First, due to its retrospective design, there was missing data in some of the variables. Second, the prediction model was not externally verified. Third, since we only examined patients from China, the model may not generalize to other populations. Future studies to validate the prediction model in various populations are warranted.

## Conclusions

This study developed and validated a nomogram to estimate OS in GBM patients that uses six prioritized variables for prognosis prediction in real-world clinical scenarios. Instead of a population-based estimate, our model provides an individualized estimate of OS based on specific patient characteristics and can be easily adopted by health care providers to counsel patients and their caregivers regarding treatment decision-making, clinical follow-up, and prognosis.

## Data Availability

The datasets used and/or analysed during the current study available from the corresponding author on reasonable request.

## Abbreviations

CPH: Cox proportional hazard
EOR: Extent of resection
EGFR: Epidermal growth factor receptor
GBM: Glioblastoma
IDH: Isocitrate dehydrogenase
KPS: Karnofsky performance status
LASSO: Least absolute shrinkage and selection operator
MGMT: O6-methylguanine-DNA methyltransferase
OS: Overall survival
PFS: Progression-free survival
TERT: Telomerase reverse transcriptase
